# Supplementary figures and images for: Induction of competent cells for Agrobacterium tumefaciens-mediated stable transformation of common bean (Phaseolus vulgaris L.)
Source: PLoS One. 2020 Mar 5;15(3):e0229909. doi: 10.1371/journal.pone.0229909 (PMC7058285; doi:10.1371/journal.pone.0229909)

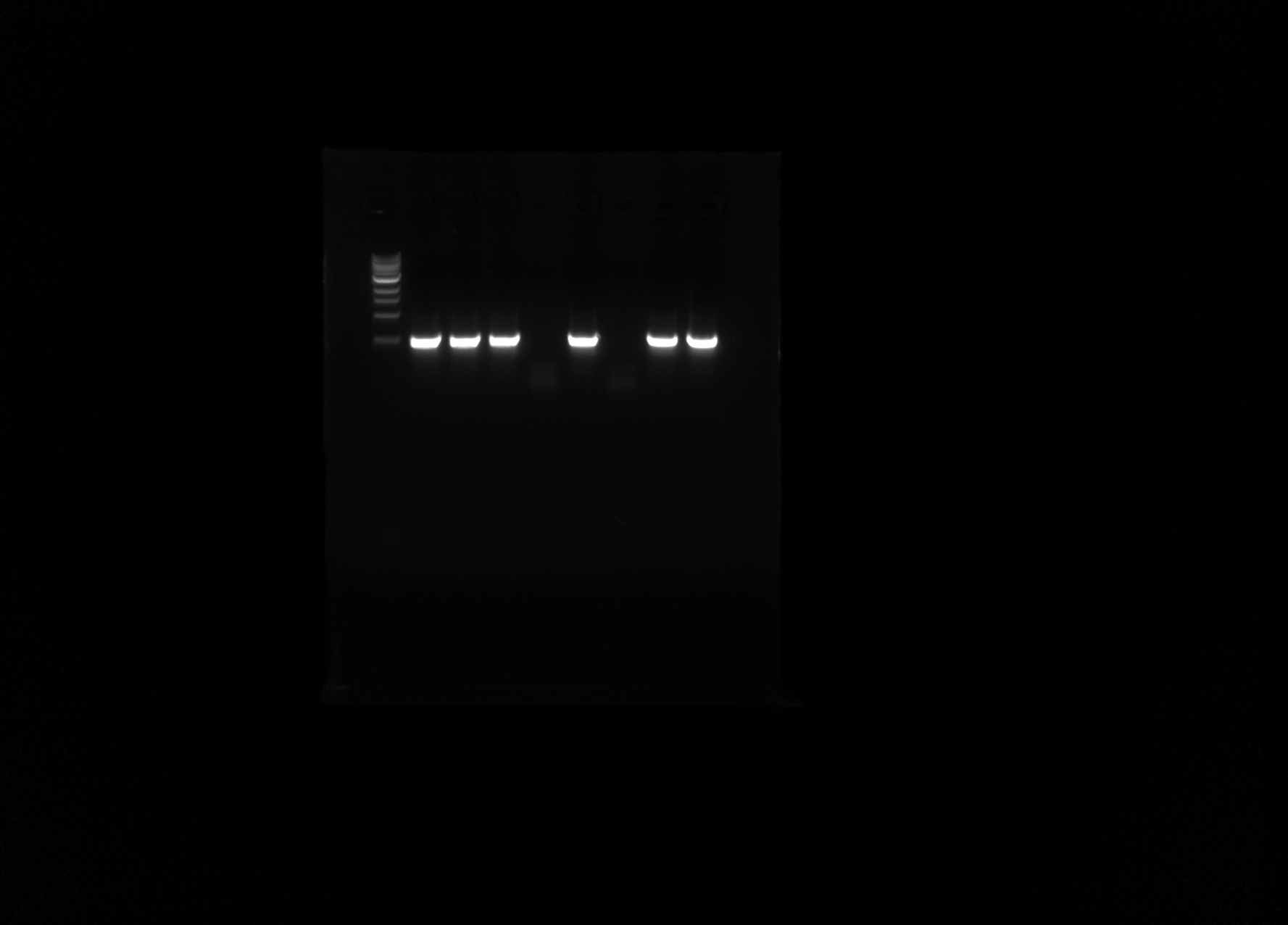

Supplement: S1 Fig — (TIF) [file pone.0229909.s002.tif]
