# Supplementary material for: Induction of competent cells for Agrobacterium tumefaciens-mediated stable transformation of common bean (Phaseolus vulgaris L.)
Source: PLoS One. 2020 Mar 5;15(3):e0229909. doi: 10.1371/journal.pone.0229909 (PMC7058285; doi:10.1371/journal.pone.0229909)
Supplement: Attachment — Submitted filename: PONE-D-19-35407-review comments.docx [file pone.0229909.s003.docx]

Dear editor and reviewers:

We would thank you for your time in reviewing our manuscript. Your review comments/suggestions are invaluable for us to further improve our manuscript. We have gone through each of them carefully and revised the manuscript to address these comments. Our response to your comments is included before for your re-review.

Reviewer #1: The paper should be deeply revised the structure of the paper according to Plos one instruction (“Results and discussions” should divided into results section and discussions section). In particular the Discussion section should be completely rewritten, should involve in “the transgenic “Embrapa 5.1” common bean for golden mosaic virus (BGMV) resistance”. In my opinion, the manuscript could not be accepted for publication in the present form.

We separated the results and the discussion into two sections. The “Embrapa 5.1” information is included in the introduction. We would think it is more reasonable to put a focus on transformation methodologies in the discussion.

1. The quality of photos (Fig.2-Fig.5, Fig. 7) is not high enough, and the label obscures the image, please replace.

High-resolution images were used to replace the Figures mentioned above.

2. The marker can't see at all in Fig.6, why? Please replace.

Replaced with a new gel picture.

3. Line 37 “This is the first stable transgenic common bean”. If you have authentic proofs please provide that. Otherwise rewrite.

The sentence was removed since it caused a misunderstanding. We meant “the first stable transgenic obtained through Agrobacterium-mediated transformation and confirmed by full genome sequencing”. The previous emphasis was not “the first stable transgenic common bean”.

4. Check the small errors in the whole manuscript (for example: Line 50 “by”, Line 93 “, and”).

Sorry about the typos and errors. We have checked the manuscript carefully to minimize these types of errors prior to this re-submission.

5. The first abbreviation shall provide the full name. For example, Line 173 “CTAB”.

Full names of the CTAB, PCR, BLAST, bp, cDNA, T-DNA were added.

6. Figure caption and citations should be referred to Plos one instruction.

Revised.

Reviewer #2: The have developed an Agrobacterium-mediated transformation protocol for Phaseolus vulgaris. Although more efficient and less time-consuming approach for Phaseolus agrobacterial transformation already exist (Collado et al., 2015), this work is worth publishing, because it presents an alternative way that might serve for gene delivery to recalcitrant Phaseolus genotypes. In general, the manuscript is well written, concise and straightforward. In my opinion this manuscript can be accepted for publishing after the authors will revise/explain following issues:

1. Authors refer to picture 7A, B or C but nothing like these letters is presented in picture 7.

Sorry about that. A new Figure 7 is included.

2. Line 293 - authors claim that T1 plants were subjected to both PCR and RT-PCR. I could not find any output of PCR analysis - it is not clear from text whether the PCR results where similar to RT-PCR or why do authors not present them in any table or image. Moreover, when presenting the RT-PCR results, the authors should show also the results of RT- (non-reverse transcribed) reaction to exclude the amplification from not properly removed genomic DNA (especially when the amplicon does not contain the intron).

Great point. We will not add the image of the PCR result using RNA samples as template. Instead, we added several sentences in lines 189-191 and 341-350 (Track-change version) to make it easier to read.

In lines 189-191

PCR analysis of the RNA samples using 35S_F and Bar_R1 primers were conducted to detect the potential DNA contamination.

In lines 341-350

In this study, a total of seven T_1_ plants, 3 and 4 for each of the two transgenic lines, were analyzed through PCR using the 35S_F and the Bar_R1 primers. The presence of the 400-bp fragment of the *bar* gene was observed in all three plants derived from transgenic line #1 and 3 out of 4 plants derived from transgenic line #2; in contrast, no fragment was detected in nontransgenic controls. The results suggested that the *bar* gene was present in six PCR-positive T_1_ plants. Similarly, expression of the *bar* gene was verified in all six PCR-positive T_1_ plants through RT-PCR using genomic DNA-free cDNA and the Bar_F and Bar_R primers (Fig 6). The PCR and RT-PCR results were consistent for the T_1_ plants.

3. Line 316 - authors claim that an integration of non-T-DNA fragment occured in T1 plants. I wonder how this can happen, despite the fact, that T1 generation plants were not subjected to Agrobacterium transformation.

In this case, both the T-TNA and its backbone region (non-T-DNA) were integrated to the genome.

5. Review Comments to the Author

Please use the space provided to explain your answers to the questions above. You may also include additional comments for the author, including concerns about dual publication, research ethics, or publication ethics. (Please upload your review as an attachment if it exceeds 20,000 characters)

Reviewer #1: The paper should be deeply revised the structure of the paper according to Plos one instruction (“Results and discussions” should divided into results section and discussions section). In particular the Discussion section should be completely rewritten, should involve in “the transgenic “Embrapa 5.1” common bean for golden mosaic virus (BGMV) resistance”. In my opinion, the manuscript could not be accepted for publication in the present form.
1. The quality of photos (Fig.2-Fig.5, Fig. 7) is not high enough, and the label obscures the image, please replace.
2. The marker can't see at all in Fig.6, why? Please replace.
3. Line 37 “This is the first stable transgenic common bean”. If you have authentic proofs please provide that. Otherwise rewrite.
4. Check the small errors in the whole manuscript (for example: Line 50 “by”, Line 93 “, and”).
5. The first abbreviation shall provide the full name. For example, Line 173 “CTAB”.
6. Figure caption and citations should be referred to Plos one instruction.

Reviewer #2: The have developed an Agrobacterium-mediated transformation protocol for Phaseolus vulgaris. Although more efficient and less time-consuming approach for Phaseolus agrobacterial transformation already exist (Collado et al., 2015), this work is worth publishing, because it presents an alternative way that might serve for gene delivery to recalcitrant Phaseolus genotypes. In general, the manuscript is well written, concise and straightforward. In my opinion this manuscript can be accepted for publishing after the authors will revise/explain following issues:

1. Authors refer to picture 7A, B or C but nothing like these letters is presented in picture 7.

2. Line 293 - authors claim that T1 plants were subjected to both PCR and RT-PCR. I could not find any output of PCR analysis - it is not clear from text whether the PCR results where similar to RT-PCR or why do authors not present them in any table or image. Moreover, when presenting the RT-PCR results, the authors should show also the results of RT- (non-reverse transcribed) reaction to exclude the amplification from not properly removed genomic DNA (especially when the amplicon does not contain the intron).

3. Line 316 - authors claim that an integration of non-T-DNA fragment occured in T1 plants. I wonder how this can happen, despite the fact, that T1 generation plants were not subjected to Agrobacterium transformation
